# Suppression of host humoral immunity by *Borrelia burgdorferi* varies over the course of infection

**DOI:** 10.1128/iai.00018-24

**Published:** 2024-03-22

**Authors:** Megan T. Williams, Yan Zhang, Mark E. Pulse, Rance E. Berg, Michael S. Allen

**Affiliations:** 1 Department of Microbiology, Immunology, and Genetics, School of Biomedical Sciences, University of North Texas Health Science Center, Fort Worth, Texas, USA; 2 The Tick-Borne Disease Research Laboratory, University of North Texas Health Science Center, Fort Worth, Texas, USA; 3 Department of Pharmaceutical Sciences, College of Pharmacy, University of North Texas Health Science Center, Fort Worth, Texas, USA; Washington State University, Pullman, Washington, USA

**Keywords:** Lyme disease, *Borrelia burgdorferi*, adaptive immune response, immune evasion, antibiotic treatment, SARS-CoV-2, immunizations

## Abstract

*Borrelia burgdorferi*, the spirochetal agent of Lyme disease, utilizes a variety of strategies to evade and suppress the host immune response, which enables it to chronically persist in the host. The resulting immune response is characterized by unusually strong IgM production and a lack of long-term protective immunity. Previous studies in mice have shown that infection with *B. burgdorferi* also broadly suppresses host antibody responses against unrelated antigens. Here, we show that mice infected with *B. burgdorferi* and concomitantly immunized with recombinant severe acute respiratory syndrome coronavirus 2 (SARS-CoV-2) spike protein had an abrogated antibody response to the immunization. To further define how long this humoral immune suppression lasts, mice were immunized at 2, 4, and 6 weeks post-infection. Suppression of host antibody production against the SARS-CoV-2 spike protein peaked at 2 weeks post-infection but continued for all timepoints measured. Antibody responses against the SARS-CoV-2 spike protein were also assessed following antibiotic treatment to determine whether this immune suppression persists or resolves following clearance of *B. burgdorferi*. Host antibody production against the SARS-CoV-2 spike protein returned to baseline following antibiotic treatment; however, anti-SARS-CoV-2 IgM remained high, comparable to levels found in *B. burgdorferi*-infected but untreated mice. Thus, our data demonstrate restored IgG responses following antibiotic treatment but persistently elevated IgM levels, indicating lingering effects of *B. burgdorferi* infection on the immune system following treatment.

## INTRODUCTION

*Borrelia burgdorferi* is a tick-borne pathogen and the major causative agent of Lyme disease (LD) in the US. First identified in the Northeastern US, case numbers and the endemic area have rapidly expanded over the past several decades (1). Recently, the Centers for Disease Control and Prevention has estimated that LD cases in the US have climbed to approximately 476,000 cases annually (2). Most LD patients return to health following antibiotic treatment. However, approximately 10%–20% of patients continue to report symptoms for a significant amount of time following treatment, a phenomenon termed post-treatment Lyme disease syndrome (PTLDS) (3–5). A recent study estimates that nearly 2 million people in the US currently live with PTLDS (6). The diagnosis and treatment of patients with PTLDS are very challenging, in large part due to the inability of current diagnostics to track disease resolution (7). However, some unique immunological signatures have been identified in PTLDS cohorts, namely, elevated CCL19 and IL-23 levels, as well as an abrogated B-cell response, potentially indicating immune dysfunction (8–10). The question of the efficacy of antibiotic treatment for LD has long been a subject of contentious debate among physicians and researchers. The primary challenge to determining if antibiotic therapy is curative is the lack of diagnostic tools to determine whether the infection has been cleared (4, 7). Some studies point to the potential for persistence of *B. burgdorferi* post-antibiotic treatment, a phenomenon that has been reported in mice (11–13), non-human primates (14–16), as well as LD patients in one clinical study utilizing xenodiagnosis (17). Additionally, one autopsy case study identified intact *B. burgdorferi* spirochetes in a patient previously infected with LD and treated with antibiotics (18). Doxycycline, the antibiotic most commonly used in treating LD in the US, is bacteriostatic, so unlike bactericidal antibiotics that kill bacterial cells, doxycycline instead inhibits the growth of *B. burgdorferi* (19). Therefore, the use of such antibiotics does, in some capacity, rely upon the patient’s immune system to clear any remaining infection. However, if *B. burgdorferi* manipulates and suppresses host immunity in a way that renders it incapable of clearing the infection, then this could potentially enable it to persist post-antibiotic treatment. This underscores the importance of studying the host immune response to *B. burgdorferi*.

*B. burgdorferi* utilizes a variety of tactics to evade and suppress the host immune response, which enable it to persist chronically. These tactics can include complement inhibition (20–26), antigenic variation (27–29), extracellular matrix degradation (30), and adaptive immune suppression (31–33). In a murine model of Lyme disease, researchers have demonstrated that *B. burgdorferi* broadly suppresses host antibody production to an unrelated antigen and induces premature germinal center (GC) collapse in the lymph nodes (LNs) approximately 1 month post-infection (31, 32). GCs are subanatomical structures within the LNs that are critical for the development of a high-affinity antibody response and the establishment of long-lived humoral immunity (34). The collapse of these structures, along with the observed abrogated antibody response against a co-administered vaccine, indicates that *B. burgdorferi* is capable of broadly suppressing host adaptive immunity through some as yet unknown mechanism. Additionally, humans previously infected with LD can be reinfected following antibiotic treatment, suggesting a lack of long-lived immunity, consistent with what has been shown in the mouse model (35, 36). However, little is known about the mechanisms utilized by *B. burgdorferi* to modulate the immune response, as well as whether these effects persist post-antibiotic treatment. Thus, further investigation of this microbial immune interference will have major implications for our current understanding of the progression of LD and will help us better understand what may drive these different clinical outcomes post-antibiotic treatment.

In the current study, we hypothesized that the host’s ability to produce antibodies against an unrelated antigen may vary over the course of infection with *B. burgdorferi* and that there may be lingering effects on the immune response following antibiotic treatment and clearance of the infection. Here we report that infection with *B. burgdorferi* continued to suppress IgG production against severe acute respiratory syndrome coronavirus 2 (SARS-CoV-2) immunization for all timepoints post-infection, although the group immunized 14 days post-infection (dpi) appeared to have the most suppressed IgG response. Additionally, following the conclusion of antibiotic treatment, the anti-SARS-CoV-2 IgG response returned to levels comparable to uninfected mice. Interestingly, anti-SARS-CoV-2 IgM levels remained unusually elevated following antibiotic treatment, similar to levels found in infected but untreated mice, indicating some lingering effects on the immune system even after clearance of the infection.

## RESULTS

### Suppression of host humoral immunity peaks 14 days post-infection

**Fig 1:**
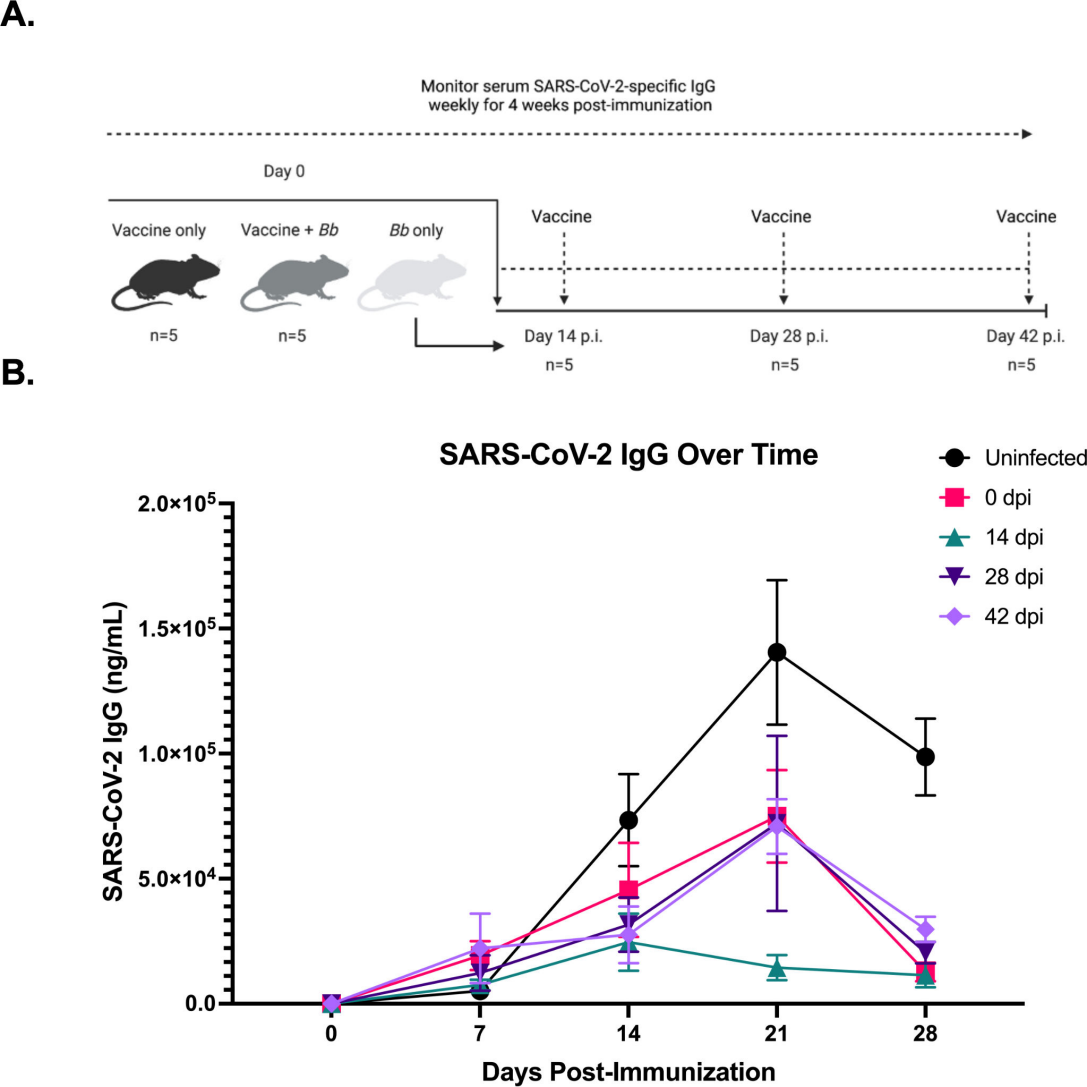
Diagram of experimental design and anti-SARS-CoV-2 IgG ± standard error of the mean (SEM) in different groups over time. (**A**) Four groups of mice (*n* = 5) were infected with *B. burgdorferi*, while one group remained uninfected. The uninfected group received the SARS-CoV-2 spike trimer immunization at day 0, and the four infected groups received the immunization at day 0, 14, 28, or day 42 post-infection, respectively. Anti-SARS-CoV-2 IgG was measured weekly for 28 days following each immunization. (**B**) All four *B. burgdorferi*-infected groups (depicted by colored lines) had significantly reduced IgG responses to the SARS-CoV-2 immunization administered at various timepoints post-infection, as denoted in the legend, compared to the uninfected, immunized control (black line, *P* = 0.0003). Significance was determined using two-way repeated-measures ANOVA followed by Dunnett’s post hoc test.

Previous studies have indicated that infection with *B. burgdorferi* results in broad suppression of host antibody responses to unrelated antigen (31). To determine how long this immune suppression lasts, we immunized mice with recombinant SARS-CoV-2 spike trimer at various timepoints post-infection and measured the SARS-CoV-2-specific IgG for 4 weeks following immunization (Fig. 1A). The SARS-CoV-2-specific IgG in the uninfected group was increased at 14 days post-immunization, peaked at 21 days, and slightly decreased at 28 days. All four *B. burgdorferi*-infected groups had reduced IgG responses against the SARS-CoV-2 immunization at 14 days post-immunization when compared with the uninfected group [analysis of variance (ANOVA) *P* = 0.0003; Dunnett’s test, *P* = 0.0111 at 21 days for the 14 dpi group vs uninfected control, *P* <0.01 at 28 days for all groups vs uninfected control]. (Fig. 1B).

**Fig 2:**
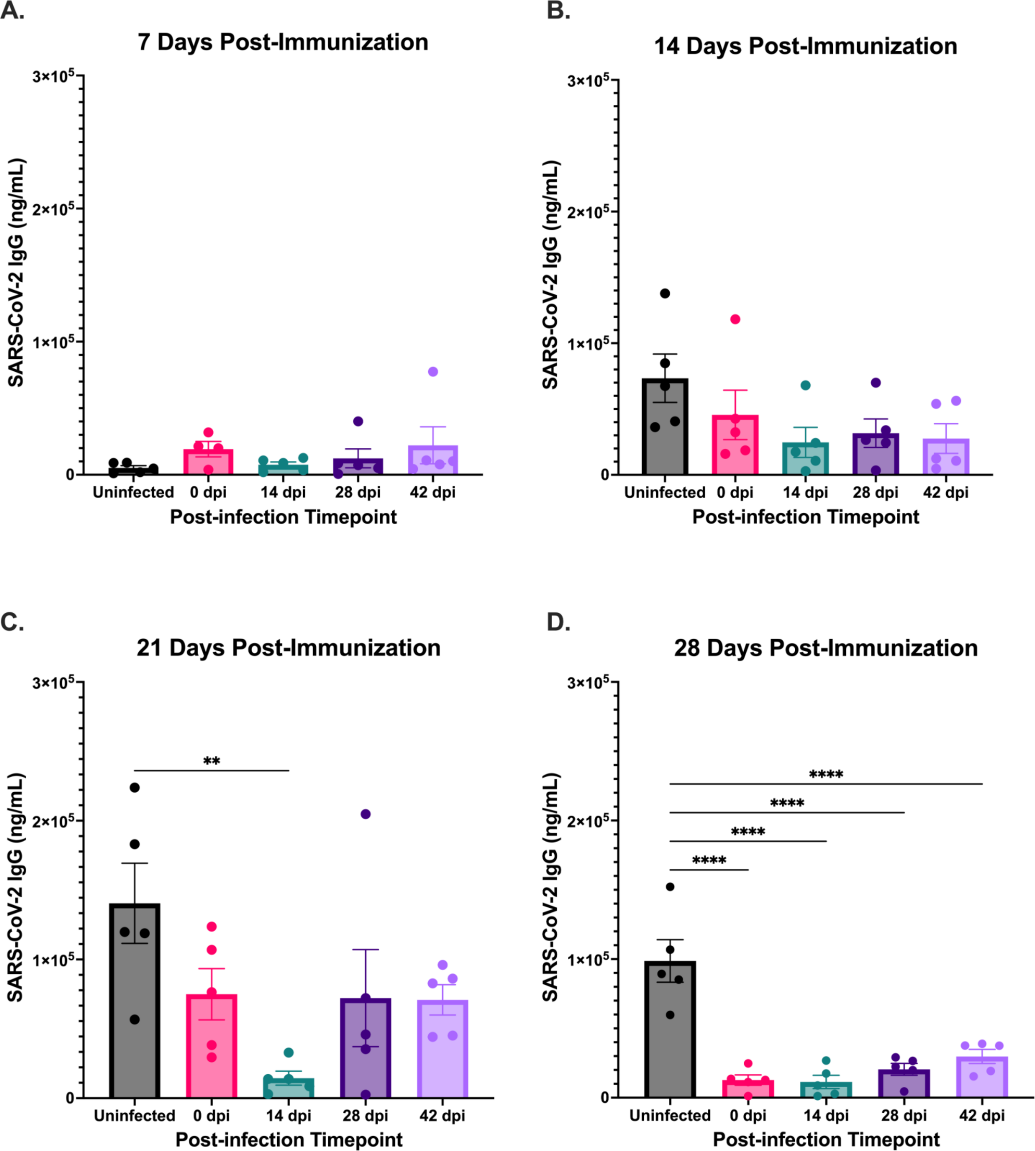
Differences in anti-SARS-CoV-2 IgG titer ± SEM between groups at (**A**) 7 days, (**B**) 14 days, (**C**) 21 days and (**D**) 28 days post-immunization with SARS-CoV-2 spike trimer. *B. burgdorferi*-infected groups (shown in colors) immunized at different timepoints post-infection and uninfected control (shown in black) are displayed. Significance was determined by one-way ANOVA followed by Dunnett’s post hoc test. **P ≤ 0.01, ****P ≤ 0.0001.

There were no significant differences in SARS-CoV-2 IgG production between the groups at 7 and 14 days post-immunization (Fig. 2A and B). Interestingly, we found that the group immunized at 14 dpi exhibited the greatest immune suppression and was the only group with a significantly (*P* = 0.0028) reduced anti-SARS-CoV-2 IgG response at 21 days post-immunization when compared to the uninfected, immunized control group (Fig. 2C). By 28 days post-immunization all *B. burgdorferi*-infected groups had significantly (*P* < 0.0001) reduced antibody responses when compared to the uninfected group (Fig. 2D). At this same timepoint, all groups of mice had robust IgG responses against *B. burgdorferi* (Fig. S1A). These results demonstrate that this broad humoral immune suppression continued for up to 10 weeks post-infection. Additionally, anti-SARS-CoV-2 IgG production was most dampened in the *B. burgdorferi*-infected group immunized 2 weeks post-infection. This may indicate that some important immunomodulatory events are occurring at this stage of the infection, which dampen the initiation of host humoral immunity.

### IgG response to SARS-CoV-2 immunization returns to normal post-antibiotic treatment

**Fig 3:**
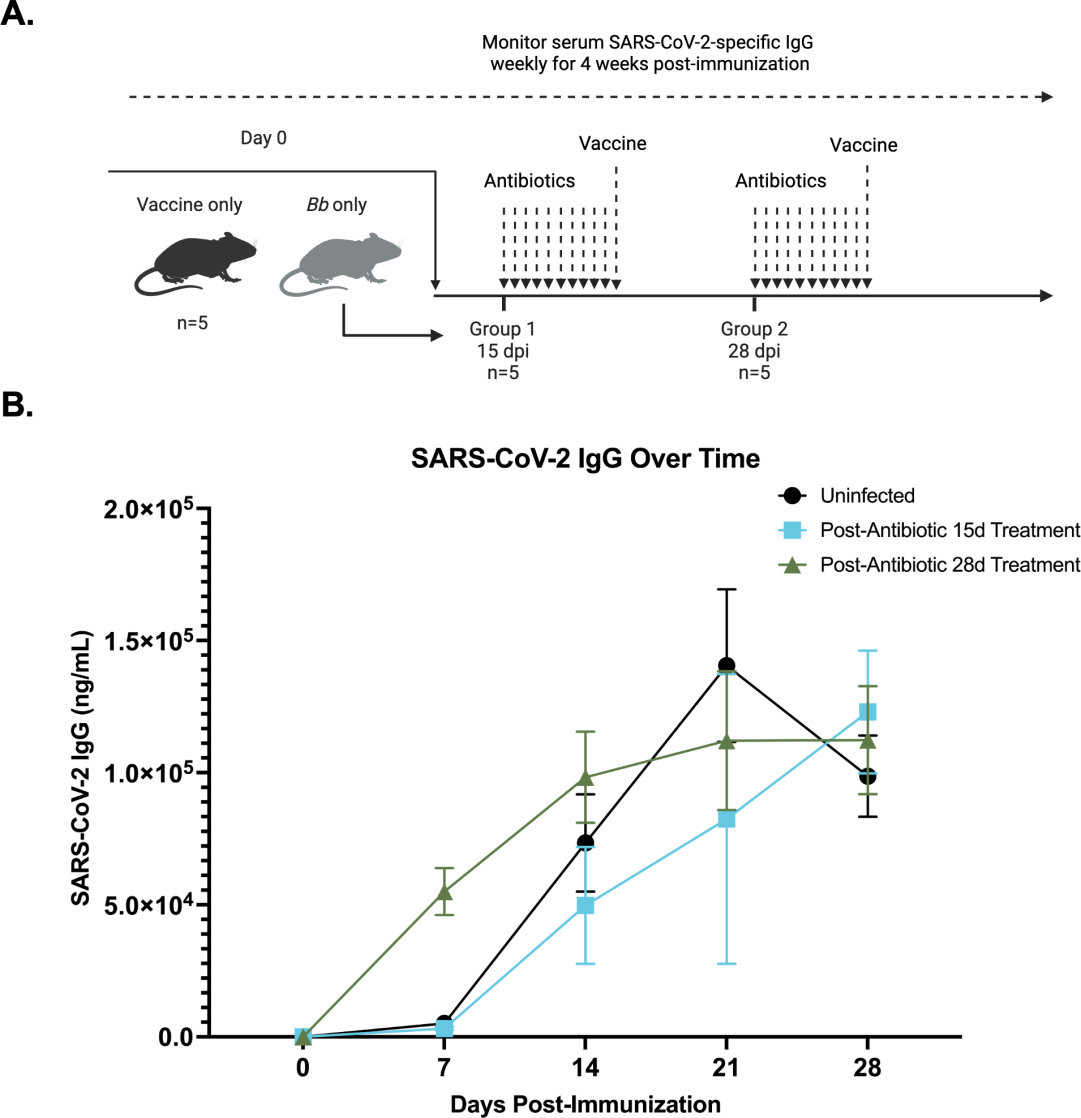
The effect of *B. burgdorferi* infection followed by antibiotic therapy on IgG production following immunization. (**A**) Diagram of experimental design and anti-SARS-CoV-2 IgG in different groups over time, in which two groups of mice (*n* = 5) were infected with *B. burgdorferi*, while one group remained uninfected. The uninfected group received the SARS-CoV-2 spike trimer immunization only at day 0. One *B. burgdorferi*-infected group began a 10-day course of 16-mg/kg ceftriaxone treatment administered intraperitoneally daily starting at 15 dpi (group 1), and the other group began treatment 28 dpi (group 2). At the conclusion of antibiotic treatment, each experimental group was immunized with the SARS-CoV-2 spike trimer. For all groups, anti-SARS-CoV-2 IgG was measured weekly for 28 days following immunization. (**B**) Neither the 15- nor 28-dpi antibiotic-treated and vaccinated group was significantly different from the uninfected control group for anti-SARS-CoV-2 IgG levels (*P* = 0.4665). Shown is the IgG concentration ± SEM. Significance was determined using two-way repeated-measures ANOVA followed by Dunnett’s post hoc test.

To further investigate the effects of humoral suppression, mice were immunized with the SARS-CoV-2 spike protein following the completion of antibiotic treatment. One group started a 10-day course of ceftriaxone at 15 dpi and the other group started treatment at 28 dpi. Immediately following the conclusion of antibiotic treatment, both groups were immunized with the SARS-CoV-2 spike protein, and serum antibody responses were monitored weekly for 4 weeks post-immunization (Fig. 3A). SARS-CoV-2 IgG responses from these treated groups were compared to the uninfected control. Neither antibiotic-treated group was significantly different from the uninfected control group (ANOVA *P* = 0.4429; Dunnett’s test, uninfected vs post-antibiotic 28-day treatment at 7 days post-immunization; *P* = 0.0075) in their production of anti-SARS-CoV-2 IgG. However, the trajectory of the IgG response appears to be slightly different with the group treated with antibiotics at 28 dpi producing significantly more anti-SARS-CoV-2 IgG at 7 days post-immunization. Overall, the humoral immune response mostly returns to baseline following clearance of infection with *B. burgdorferi* (Fig. 3B).

**Fig 4:**
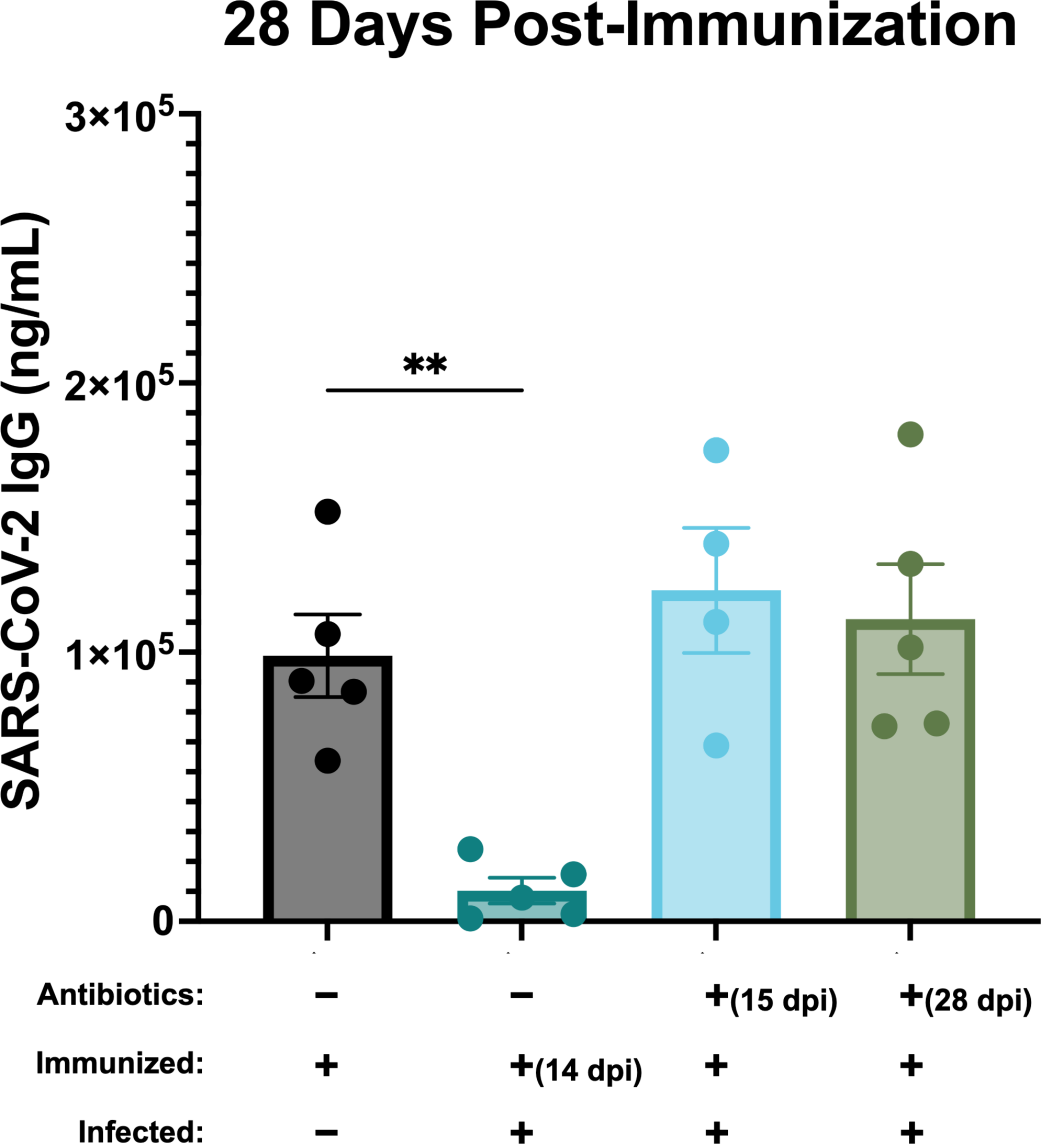
IgG response ± SEM to unrelated antigen in uninfected, *B. burgdorferi*-infected, and *B. burgdorferi*-infected antibiotic-treated groups 4 weeks post-immunization. Post-infection timepoints for immunization and antibiotic treatment are denoted when applicable. Shown are differences between the uninfected and immunized group, *B. burgdorferi*-infected groups that were immunized at day 0 post-antibiotic treatment, and the *B. burgdorferi*-infected but untreated group that was immunized at 14 dpi. Significance was determined by one-way ANOVA followed by Dunnett’s post hoc test. **P≤0.01.

Because the timepoint 28 days post-immunization is where the greatest significant difference was observed between *B. burgdorferi*-infected and uninfected groups previously (Fig. 2D), we chose to compare these groups with the antibiotic-treated groups at the same timepoint. At 4 weeks post-immunization, there was no difference in anti-SARS-CoV-2 IgG production between antibiotic-treated and uninfected groups (Fig. 4). Correspondingly, both antibiotic-treated groups had significantly higher SARS-CoV-2 IgG production when compared with the most suppressed *B. burgdorferi*-infected group that was immunized at 14 dpi (Fig. 4). However, the IgG response against *B. burgdorferi* was significantly reduced 28 days post-antibiotic treatment, indicating a lack of long-lived immunity (Fig. S1A). Additional data comparing SARS-CoV-2 IgG production among the *B. burgdorferi*-infected, uninfected, and antibiotic-treated groups at 7 days (Fig. S2), 14 days (Fig. S3), and 21 days (Fig. S4) post-immunization can be found in the supplemental material.

### Infection with *B. burgdorferi* increases IgM production against an unrelated antigen, and this effect persists following antibiotic treatment

**Fig 5:**
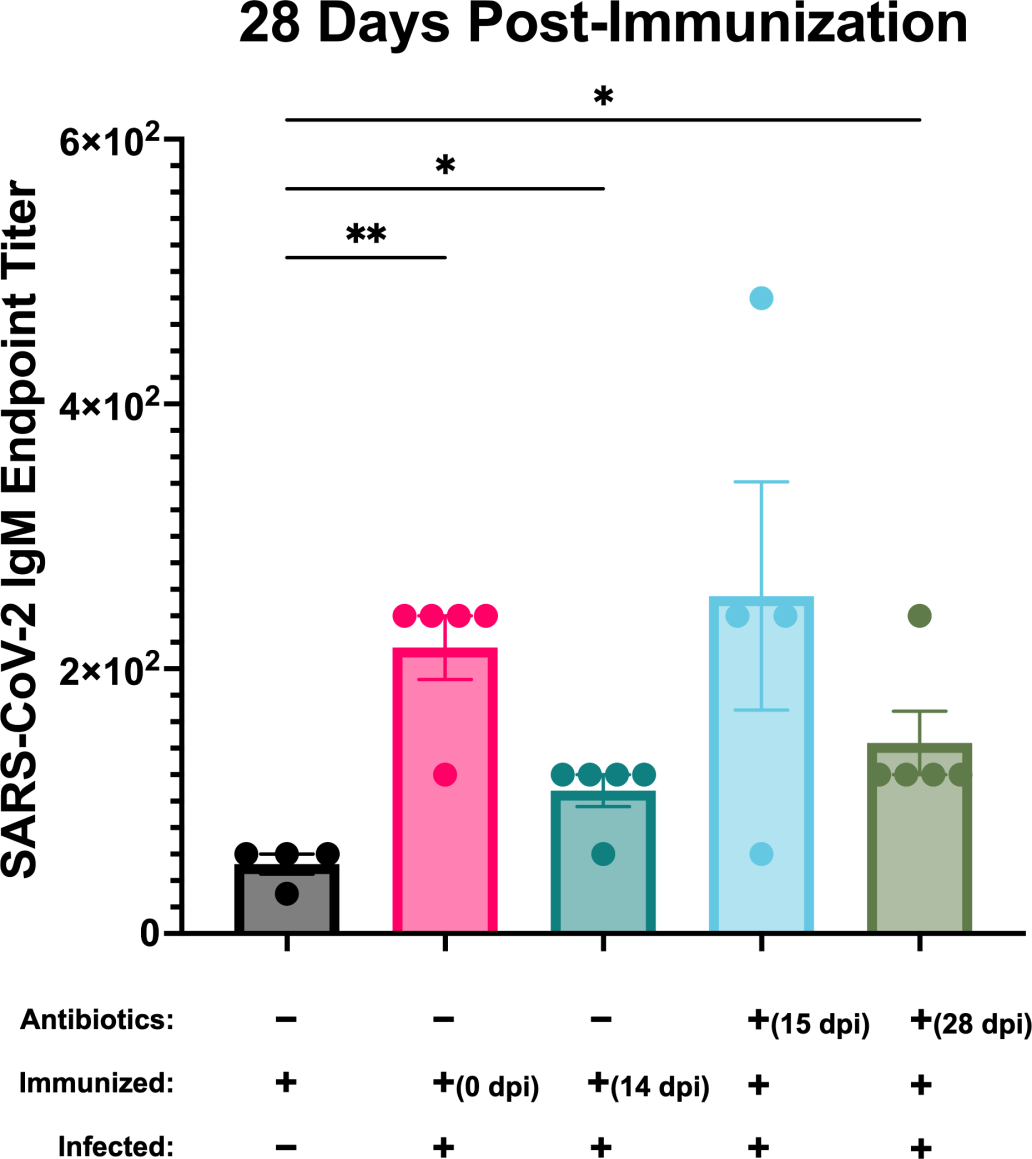
IgM response to SARS-CoV-2 immunization in uninfected (black), *B. burgdorferi*-infected (pink and teal), and previously infected, antibiotic-treated groups (light blue and green). Post-infection timepoints for immunization and antibiotic treatment are denoted when applicable. Shown are the endpoint titer means ± SEMs using a cutoff of Abs_450_ = 0.2. Significance was determined by Welch’s ANOVA followed by Dunnett’s post hoc test. *P ≤ 0.05, **P ≤ 0.01.

Infection with *B. burgdorferi* is characterized by an unusually strong and persistent IgM production (32, 37). A recent study demonstrated that *B. burgdorferi* infection results in an exaggerated IgM response against an unrelated antigen (38), indicating that *B. burgdorferi* actively modulates the immune response toward IgM production. *B. burgdorferi*-infected mice immunized at 0 and 14 dpi, as well as mice treated with antibiotics at 28 dpi produced significantly more (*P* = 0.0044, 0.0269, and 0.049, respectively) anti-SARS-CoV-2 IgM when compared with the uninfected, immunized control group (Fig. 5). IgM production also appears to be elevated in the group treated with antibiotics 15 days post-infection, although these levels did not reach statistical significance. Likewise, IgM specific for *B. burgdorferi* remained elevated in the two antibiotic-treated groups, with levels comparable to actively infected mice (Fig. S1B). Taken together, we found that, although the IgG response to the unrelated SARS-CoV-2 antigen returned to normal following antibiotic treatment, the IgM response interestingly did not.

### Lymph node structure returns to normal following antibiotic treatment

**Fig 6:**
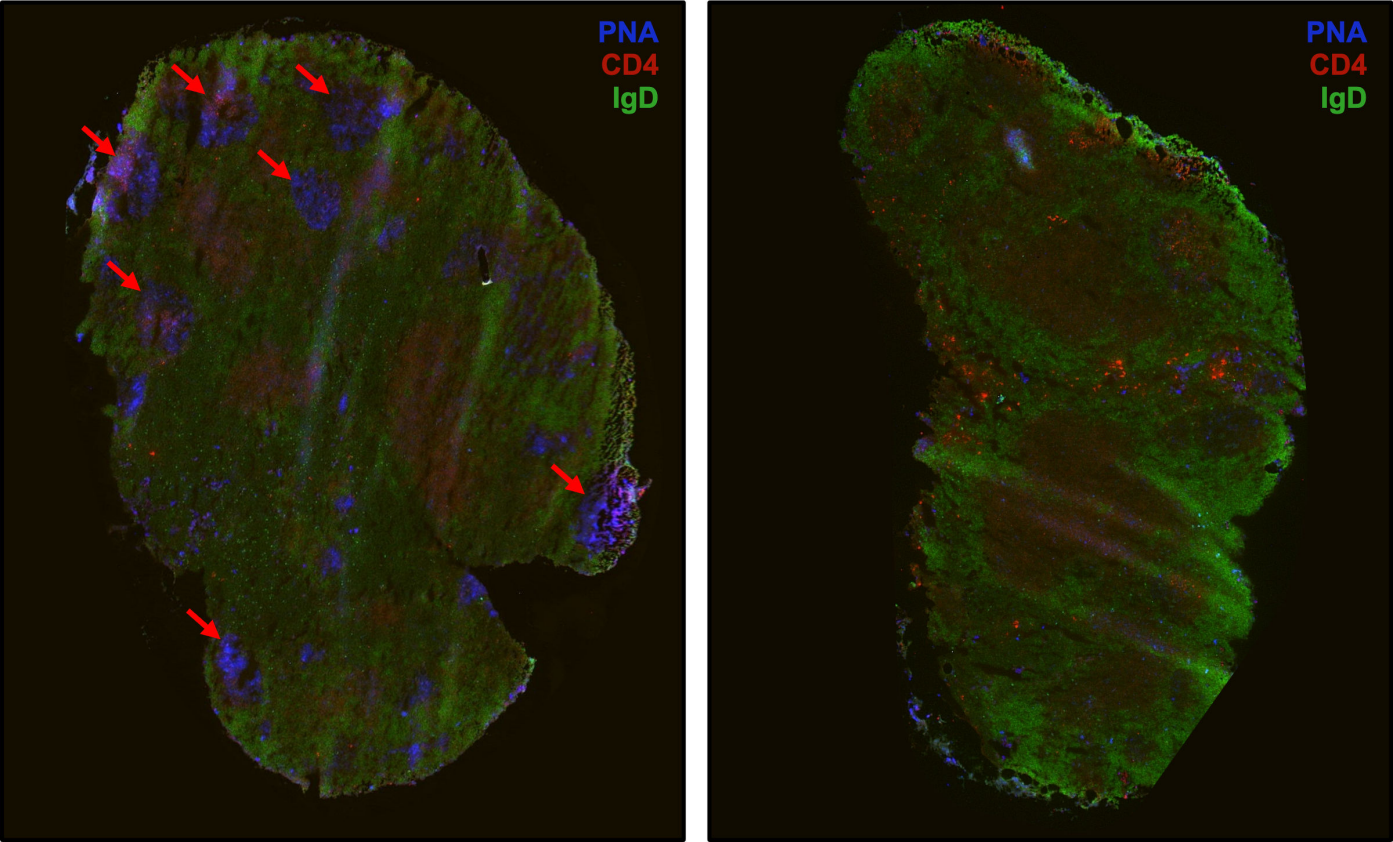
Immunofluorescent staining of right inguinal lymph nodes at 15 days post-infection (left) and 30 days post-infection (right). Shown is one representative set of sections. IgD staining indicates B-cell follicles (IgD+, green); PNA staining indicates GCs (PNA+, blue); and CD4 staining indicates helper T cells (CD4+, red). Arrows indicate GCs.

Previous studies have shown that beginning at around 10 days post-infection with *B. burgdorferi*, lymphoid architecture is disrupted, characterized by massive accumulation of naïve B cells and the deterioration of distinct T- and B-cell zones (32). It has also previously been shown that GCs collapse approximately 1 month post-infection with *B. burgdorferi* (31, 32). Here we confirm this finding using a different method of infection. Needle inoculation was used in the current study compared to the tissue-adapted spirochete infection model utilized in previous studies cited here (31, 32, 37). Germinal centers are visible 15 days post-infection and undetectable 30 days post-infection (Fig. 6). There is also a massive accumulation of B cells 15 days post-infection and a lack of clearly defined T-cell zones consistent with previous findings (37).

**Fig 7:**
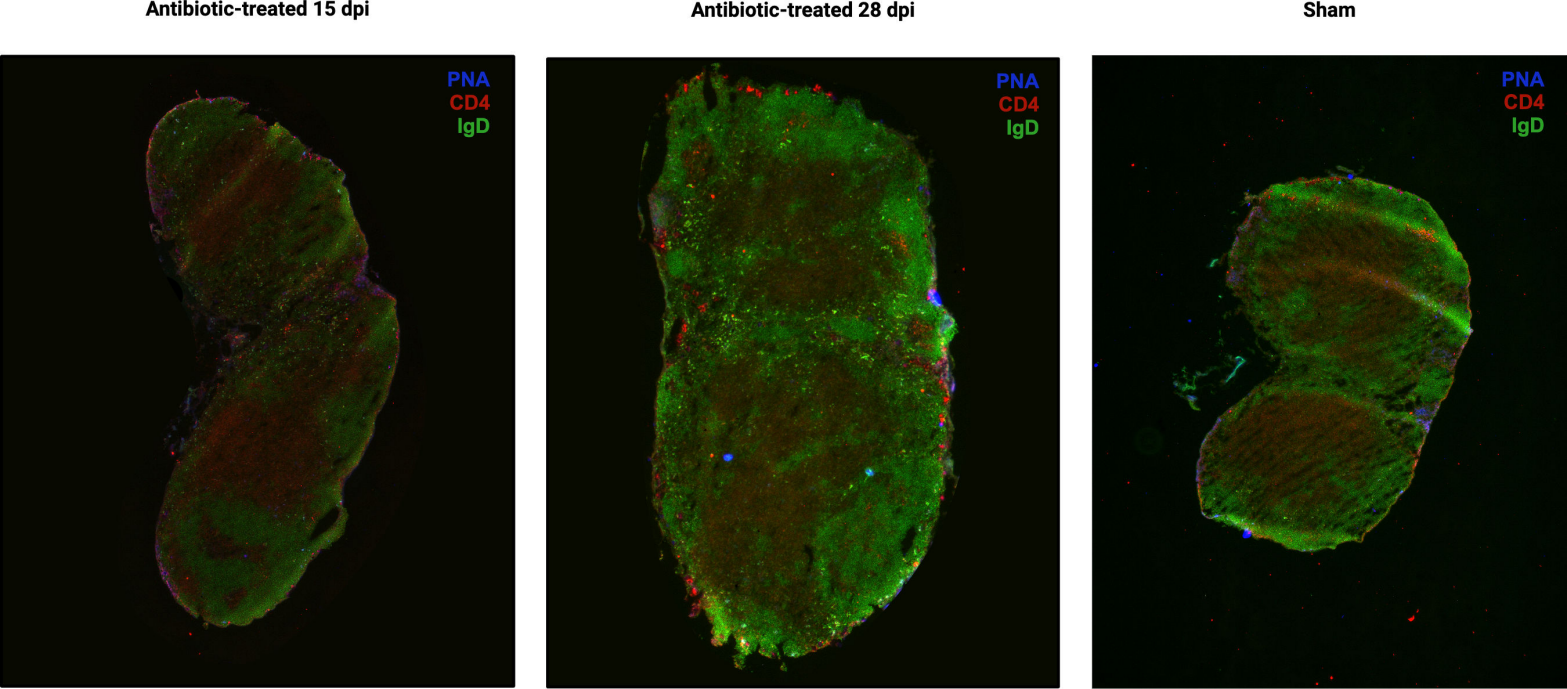
Immunofluorescent staining of right inguinal lymph nodes from SARS-CoV-2 immunized mice 4 weeks post-antibiotic treatment beginning at 15 dpi (left) and 28 dpi (center), respectively. Lymphoid structure in antibiotic-treated mice is compared to lymph node from sham-infected (phosphate-buffered saline) mouse (right). IgD staining indicates B-cell follicles (IgD+, green); PNA staining indicates GCs (PNA+, blue); and CD4 staining indicates helper T cells (CD4+, red). There are no visible GCs.

Following antibiotic treatment, lymph node structure returns to normal and is comparable to the sham control (Fig. 7). T- and B-cell zones appear to be more defined in antibiotic-treated mice with a typical follicular structure.

## DISCUSSION

Infection with *B. burgdorferi* requires antibiotic treatment for resolution (39). Without treatment, *B. burgdorferi* can persist in the mammalian host by utilizing a variety of immune evasion and suppression mechanisms, enabling its success in the enzootic cycle (40). Previous studies have shown that mice infected with *B. burgdorferi* fail to establish long-lived humoral immunity against the pathogen and are unable to respond productively to a co-administered influenza vaccine (31). This raises the question of how long these effects last and whether the immune system returns to normal following antibiotic treatment. This is clinically relevant, considering the vast majority of Lyme disease patients do receive antibiotic intervention. Data presented in this article indicate that humoral immune suppression continues for at least 10 weeks post-infection, our last timepoint measured (Fig. 1B). Here we used a SARS-CoV-2 spike protein immunization which has been previously used (41) to detect humoral immune suppression. Interestingly, a study conducted in Poland found that increased levels of serum *Borrelia*-specific IgG was strongly correlated with COVID-19 severity and risk of hospitalization (42), suggesting the potential for compromised immunity in former Lyme disease patients. However, it remains unclear whether the patients included in that study tested seropositive due to active infection or due to prior exposure to *Borrelia*. Further studies should be done to elucidate these differences.

Interactions between B cells and T follicular helper cells (T_FH_) in the GCs are very important for the generation of high-affinity antibodies and long-lived humoral immunity (43–45), both of which fail to be properly established during infection with *B. burgdorferi* (31). Interestingly, studies have previously shown that GC-positioned T_FH_ cells and B cells in the LNs peak approximately 2 weeks post-infection and then rapidly decline thereafter (31). This 2-week timepoint coincides with the peak humoral immune suppression observed in the present study. Here we found that mice infected with *B. burgdorferi* and immunized 2 weeks post-infection had the most suppressed IgG response against the immunization out of all groups tested (Fig. 2C and D). These data suggest that this timepoint may be a critical point in the infection, with events occurring that eventually lead up to, and potentially cause, germinal center collapse in the lymph node, resulting in broad humoral immune suppression and dysfunction. More work needs to be done to better understand what host-pathogen interactions may be occurring at this timepoint that may result in the immune dysfunction observed. Additionally, it is possible that the suppressed response to the SARS-CoV-2 immunization shown here may be due, at least in part, to the humoral immune response being redirected toward fighting the active infection with *B. burgdorferi*. We did detect a robust IgG response to *B. burgdorferi* during active infection, but this response failed to be maintained following the conclusion of antibiotic treatment, suggesting a lack of long-lived humoral immunity (Fig. S1A), consistent with previous findings (31). It has also been previously shown that the humoral immune response against *B. burgdorferi* is characterized by predominantly T-independent B-cell responses and a failure to produce long-lived antibodies against T-dependent *Borrelia* antigens (31). This could also possibly explain the findings in the present study, as immunoglobulin class switching typically requires interactions between T and B cells (46). Additionally, the *Borrelia*-specific IgM response is comparable before and after antibiotic treatment, similar to the trends observed in the anti-SARS-CoV-2 IgM response (Fig. S1B). Future research should work to further disentangle the infection-specific and immunization-specific responses so that we can better understand what may be causing the effects on the immune system observed.

Although it has been demonstrated that active infection with *B. burgdorferi* broadly suppresses host IgG responses against a co-administered vaccine, it was previously unclear whether these effects persisted following antibiotic treatment. Here we demonstrate that the IgG response against a SARS-CoV-2 immunization is restored post-antibiotic treatment (Fig. 4). This indicates that upon clearance of the active infection, the immune system returns to a normal state, which suggests that modulation of host immunity requires the presence of live, viable *B. burgdorferi* spirochetes. It is important to note that ceftriaxone was used in the present study, a bactericidal antibiotic, rather than doxycycline, a bacteriostatic antibiotic, which is most commonly prescribed in human Lyme disease patients (19). Although tetracyclines, such as doxycycline, are commonly used to treat human Lyme disease patients, their efficacy in infected mice is poor (47). It may be of interest in future studies to see if treatment with doxycycline would generate similar results. Additionally, in the present study, mice were immunized with the SARS-CoV-2 spike protein immediately following the conclusion of antibiotic treatment. It has been shown previously that a small percentage of mice still test positive for live *B. burgdorferi* infection through xenodiagnosis 1–3 months post-antibiotic treatment (12). In the current study, samples were collected only up to 1 month post-antibiotic treatment. Although all tissue biopsies tested were culture negative, it is possible that if the mice were given more time, some may have shown a resurgence in infection later on (13). In future studies, it would be interesting to allow more time to pass between the end of the antibiotic course and the immunization. Mice persistently infected with *B. burgdorferi* might again exhibit humoral immune suppression at these later timepoints post-antibiotic treatment.

Although the anti-SARS-CoV-2 IgG response was restored following antibiotic treatment, anti-SARS-CoV-2 IgM and anti-*Borrelia* IgM remained elevated, comparable to that of actively infected mice (Fig. 5; Fig. S1B). According to a recent study, *Borrelia*-specific IgM controls blood-borne bacteremia but not bacterial dissemination (38). This is thought to be due to the size of pentameric IgM, making it less able to extravasate into tissues, especially immunologically privileged sites, where *B. burgdorferi* tends to be found (48). Therefore, *B. burgdorferi* may skew the humoral immune response away from IgG production and toward IgM production for its own benefit, decreasing the likelihood of successful clearance of the infection. It was previously shown that *B. burgdorferi* induces exaggerated IgM production against an unrelated antigen, indicating that IgM production is the product of active manipulation of host immunity and not simply due to the nature of borrelial proteins (38). Here, we show that this effect may continue following antibiotic treatment. Exaggerated IgM responses have also been reported in human Lyme disease patients, even for long periods of time following antibiotic treatment (49, 50). One clinical study found that 38% of patients who previously received antibiotic treatment for Lyme disease still tested positive on an IgM immunoblot 1 year after the initial infection (49). Another study found that serum IgM against *B. burgdorferi* can persist in patients 10–20 years following the initial infection (50). It is unclear at what point post-antibiotic treatment this IgM production returns to baseline and what accounts for the variability from patient to patient. Future studies should be conducted to further elucidate this and to better understand how *B. burgdorferi* drives this response, and whether the long-term production of IgM is to the benefit or detriment of the host.

Immunofluorescent staining of the nearest draining lymph nodes confirmed the presence of germinal centers at 15 days post-infection and the absence of germinal centers at 30 days post-infection (Fig. 6) as has been previously shown (31). There was also weak CD4+ T-cell staining at 15 days post-infection and greatly increased IgD+ B-cell staining compared to 30 days post-infection, indicating a massive expansion of naïve B cells. Lymphoid structure was disorganized at 15 days post-infection with a deterioration of defined T- and B-cell zones. Lymph node structure appears to return to baseline following antibiotic treatment and is comparable to lymph nodes from sham-infected mice (Fig. 7). This indicates that the early disorganization of lymphoid structure is likely due to the presence of live *B. burgdorferi*, and continued dysfunction in the lymph node requires continued infection.

In conclusion, the data presented here demonstrate that suppression of host IgG responses against an unrelated antigen peaks 2 weeks post-infection and require the presence of live *B. burgdorferi*, but exaggerated IgM production against the same antigen may persist following the clearance of *B. burgdorferi* with antibiotics. This may indicate that infection with *B. burgdorferi* results in at least some lasting effects on host immunity even after antibiotic treatment, although the mechanisms underpinning this and the implications it may have on the competency of the immune system remain unclear.

## MATERIALS AND METHODS

### Mice

Female 6- to 8-week-old C57BL/6J (B6) mice were purchased from The Jackson Laboratory (Bar Harbor, ME). All mice were maintained in microisolator cages under specific pathogen-free conditions in the University of North Texas Health Science Center, AALAC-accredited Department of Lab Animal Medicine BSL-2 facility.

### Infections with *Borrelia burgdorferi* and antibiotic treatment

*B. burgdorferi sensu stricto* strain N40 (kindly provided by Dr. Monica Embers at Tulane National Primate Research Center) was cultured in modified Barbour-Stoenner-Kelley H medium at 37°C and 5% CO_2_ to the mid-log phase. The inoculum was prepared as previously described (51). In short, *B. burgdorferi* was washed three times and resuspended in cold 50% rabbit serum in phosphate-buffered saline (PBS), enumerated with a Petroff-Hauser bacterial cell counting chamber (Baxter Scientific), and diluted to 10^6^ spirochetes/mL. Mice were injected subcutaneously on the right hind leg region with 10^5^ spirochetes. Sham-infected mice were injected in the same way with 50% rabbit serum in PBS. All infections were confirmed through culture, as previously described (52), and through PCR detection of *B. burgdorferi* in tissue samples collected at necropsy (Table S1). When stated, *B. burgdorferi*-infected mice were treated once daily for 10 days with 16-mg/kg ceftriaxone (C5793; Sigma, St. Louis, MO) in 100-µL PBS injected intraperitoneally as previously described (31). Prior to antibiotic treatment, ear punch biopsies were collected from mice so that infection could be confirmed using both PCR and culture methods. Ear punch biopsies from all mice in the group treated 28 days post-infection tested positive. However, detection of *B. burgdorferi* in ear punch biopsies from the mice treated 15 days post-infection was highly variable (Table S2). This is likely due to it being an early timepoint in the infection, and the rate of dissemination from the injection site (tail base) to the ear is variable from mouse to mouse. However, all mice from this group were serologically positive and had comparable antibody responses against *B. burgdorferi* to the other antibiotic-treated group. At the study endpoint post-antibiotic treatment, tissues were collected and cultured for the presence of *B. burgdorferi* to confirm clearance of the infection as previously described (52).

### SARS-CoV-2 immunizations

Recombinant SARS-CoV-2 spike trimer was purchased from R&D Systems (cat #10549-CV; R&D Systems, Minneapolis, MN), and AddaVax oil-in-water squalene emulsion was purchased from Invivogen (Toulouse, France). Mice were randomized to different groups and given a single immunization subcutaneously at the nape with 5 µg of recombinant SARS-CoV-2 trimer administered in 200 µL of 50% AddaVax oil-in-water squalene emulsion in PBS as described previously (41).

### SARS-CoV-2 IgG and IgM ELISA

Immulon 4HBX plates (Thermo Scientific, Waltham, MA) were coated overnight with 0.05-µg/well recombinant SARS-CoV-2 trimer (R&D Systems) at 4°C. After blocking with 3% bovine serum albumin (BSA), serum and standard were added in PBS with 2% BSA and incubated for 2 h at room temperature (RT). Goat anti-mouse IgG conjugated to horseradish peroxidase (HRP) (cat #A16084; Invitrogen, Waltham, MA) or goat anti-mouse IgM conjugated to HRP (cat #62–6820, Invitrogen) were diluted 1:5,000 or 1:2,000, respectively, in 1% BSA and incubated for 1 h at RT. After incubation, antigen-specific antibodies were detected using TMB (3,3',5,5'-tetramethylbenzidine) substrate as described by the manufacturer (Thermo Scientific), and the reaction was stopped with 2-M H_2_SO_4_. A standard curve for the IgG enzyme-linked immunosorbent assay (ELISA) was generated using a SARS-CoV-2 spike S1 subunit antibody (cat #MAB105403, R&D Systems) starting with a 500-ng/mL concentration. Only standard curves with an *R*^2^ value greater than 0.9 were used to determine the concentration of the anti-SARS-CoV-2 IgG. The lower and upper limits of quantification for this assay were 4.844 and 79.712 ng/mL, respectively. Absorbance was measured at 450 nm using a BioTek Synergy 2 microplate reader (BioTek, Winooski, VT). Antibody endpoint titers for the IgM ELISA were determined as the highest serum dilution corresponding to a cutoff of 0.2 Abs_450_.

### *Borrelia burdorferi* IgG and IgM ELISA

*B. burgdorferi* whole-cell lysate was prepared as previously described (53). Total protein in the sonicate was quantified on the basis of the absorbance at 280 nm using a NanoDrop1000 spectrophotometer (Thermo Scientific), and aliquots were stored at −20°C. The protein was diluted to 5 µg/mL, and 50 µL per well was placed in Immulon 4HBX plates (Thermo Scientific) and coated overnight at 4°C. After blocking with 3% BSA, serially diluted serum was added in PBS with 2% BSA and incubated for 2 h at RT. Goat anti-mouse IgG conjugated to HRP (cat #A16084, Invitrogen) or goat anti-mouse IgM conjugated to HRP (cat #62–6820, Invitrogen) was diluted to 1:5,000 or 1:2,000, respectively, in 1% BSA and incubated for 1 h at RT. After incubation, antigen-specific antibodies were detected using TMB substrate as described by the manufacturer (Thermo Scientific), and the reaction was stopped with 2-M H_2_SO_4_. Antibody endpoint titers for IgG were determined as the highest serum dilution corresponding to a cutoff of 1.0 Abs_450_. Antibody endpoint titers for IgM were determined as the highest serum dilution corresponding to a cutoff of 0.2 Abs_450_.

### DNA extraction and PCR

Murine tissue samples were harvested and stored at −80°C until processing. DNA from tissue samples were extracted using the DNeasy Blood and Tissue Kit (Qiagen, Hilden, Germany) following a protocol kindly provided by Dr. Jenny Hyde at Texas A&M Health Science Center. In short, tissue samples were digested in 1% collagenase and proteinase K for 2–3 h, and then DNA was extracted according to the manufacturer’s protocol. Extracted DNA was eluted to an adjusted final volume of 100 µL, quantified using a NanoDrop1000 spectrophotometer (Thermo Scientific), and stored at −20°C.

Presence or absence of *B. burgdorferi* was detected using the following primers used previously (54), targeting *recA*: forward (5′-GTGGATCTATTGTATTAGATGAGGCTCTCG-3′) and reverse (5′-GCCAAAGTTCTGCAACATTAACACCTAAAG-3′). The PCR cycles were carried out with an initial denaturation step for 5 min at 94°C; 40 cycles of denaturation for 15 s at 94°C, annealing for 30 s at 60°C, and extension for 20 s at 68°C; and a final extension step at 68°C for 5 min. Each reaction was conducted in a total volume of 25 µL containing 2.5-µL 10× Accuprime PCR Buffer II (Invitrogen, Carlsbad, CA), 2.5-µL 10× bovine serum albumin, 1.0-µL Mg^2+^ (Invitrogen), 0.5-µL forward and reverse primers (10 µM), 0.1 µL of Accuprime Taq DNA Polymerase High Fidelity (5 U/µL), 1 µL of template DNA (10–100 ng), and 16.9-µL molecular-grade water. PCRs were performed in a thermocycler (Bio-Rad, Hercules, CA). Positive control (DNA extracted from *B. burgdorferi* N40 culture) and negative control (PCR master mix) were used in each PCR run.

### Immunofluorescence

The nearest draining lymph node (right inguinal) was harvested, snap frozen, embedded in Tissue-Tek OCT media (Sakura, Torrance, CA) and stored at −80°C. Lymph nodes were cut into 5-μm-thick sections on a cryostat (Leica, Wetzlar, Germany) and dried onto Superfrost/Plus slides (Fisher Scientific, Waltham, MA) for 2 h at RT. Slides were fixed and permeabilized in ice-cold acetone for 10 min. Immunofluorescent staining was done as previously described (55). In short, slides were washed and then blocked with 3% BSA for 2 h at RT. Slides were then washed and blocked with streptavidin and biotin to eliminate endogenous biotin activity and then stained overnight at 4°C with CD4-AlexaFluor647 (clone EPR19514; Abcam, Cambridge, UK), IgD-FITC [clone 11–26c (11–26), eBioscience, Waltham, MA], and PNA-Biotin (Vector Labs, Newark, CA). Slides were then washed and stained for 1–2 h at room temperature with streptavidin-AlexaFluor350 (Invitrogen). Slides were then washed and mounted in Fluoromount-G (SouthernBiotech, Birmingham, AL). Slides were imaged with the Keyence AIO fluorescence microscope (Keyence, Osaka, Japan).

### Statistical analysis

All graphs were prepared, and statistical analysis was performed with GraphPad Prism version 10.0.2 software (GraphPad Software, La Jolla, CA). Schematic diagrams were created using BioRender (BioRender, Toronto, Canada).

## SUPPLEMENTAL MATERIAL

Supplemental material iai.00018-24-s0001.docx Fig. S1 to S4; Tables S1 and S2.

ASM does not own the copyrights to Supplemental Material that may be linked to, or accessed through, an article. The authors have granted ASM a non-exclusive, world-wide license to publish the Supplemental Material files. Please contact the corresponding author directly for reuse.
