# Supplementary material for: Suppression of host humoral immunity by Borrelia burgdorferi varies over the course of infection
Source: Infect Immun. 2024 Mar 22;92(4):e00018-24. doi: 10.1128/iai.00018-24 (PMC11003232; doi:10.1128/iai.00018-24)
Supplement: Supplemental material — Fig. S1 to S4; Tables S1 and S2. [file iai.00018-24-s0001.docx]

**Suppression of Host Humoral Immunity by *Borrelia burgdorferi* Varies Over the Course of Infection**

**Megan T. Williams^1*^, Yan Zhang^1^, Mark Pulse^2^, Rance E. Berg^1^, Michael S. Allen^1*^**

^*^Correspondence:

Megan Williams & Michael Allen

Meg.t.will@gmail.com (M.T.W), Michael.Allen@unthsc.edu (M.S.A)

# Supplementary Figures


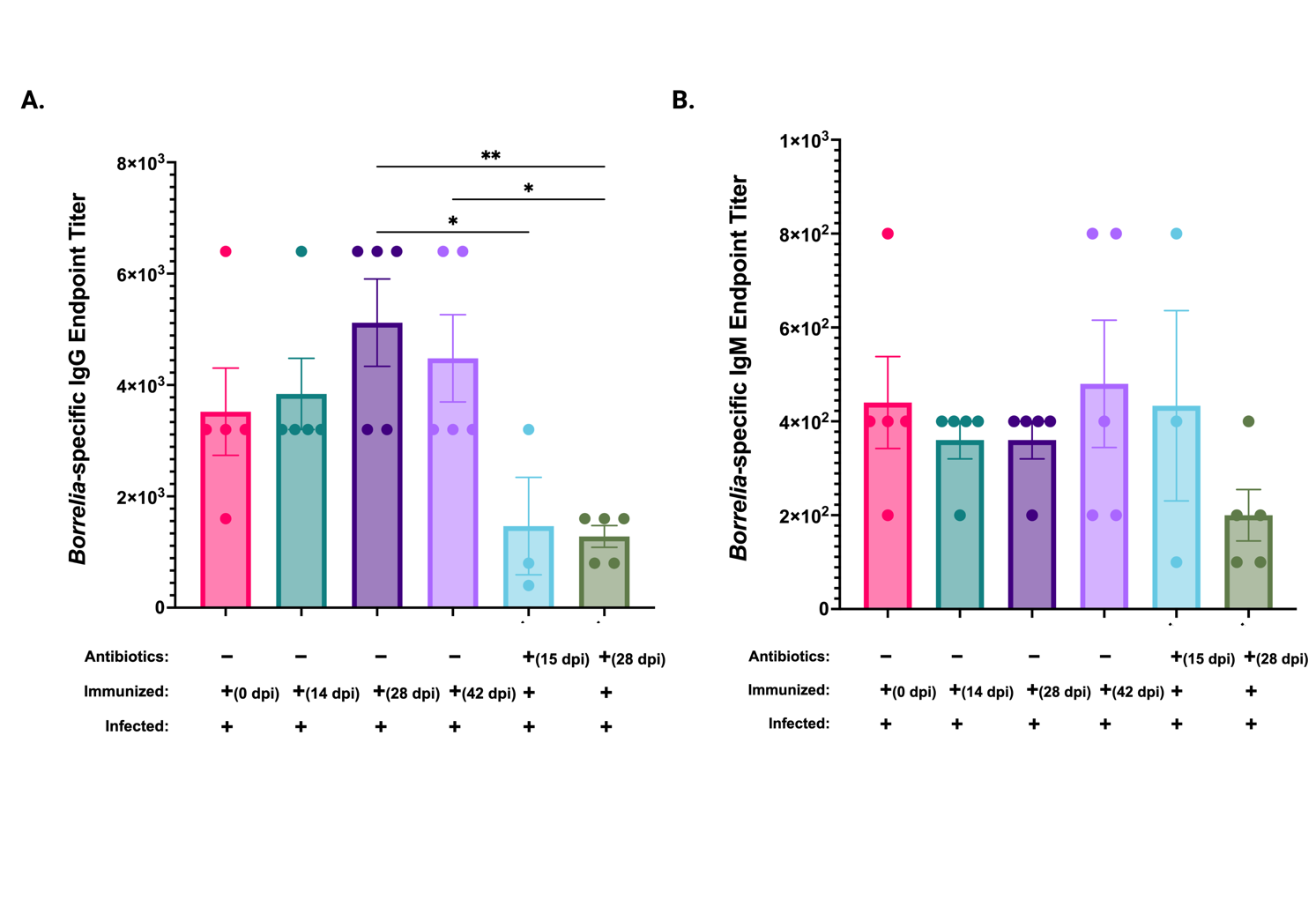


**Figure S1.** IgG response (A) and IgM response (B) ± SEM to *B. burgdorferi* in infected but untreated, and *B. burgdorferi*-infected antibiotic-treated groups 28 days post-immunization with the SARS-CoV-2 spike protein. Post-infection timepoints for immunization and antibiotic treatment are denoted when applicable and timepoint since the start of infection is also denoted to aid in data interpretation. Titers from uninfected mice did not meet the threshold of detection. Shown are differences between all groups and significance was determined by one-way ANOVA followed by a Tukey post-hoc test (**p$\leq$0.01, *p$\leq$0.05).


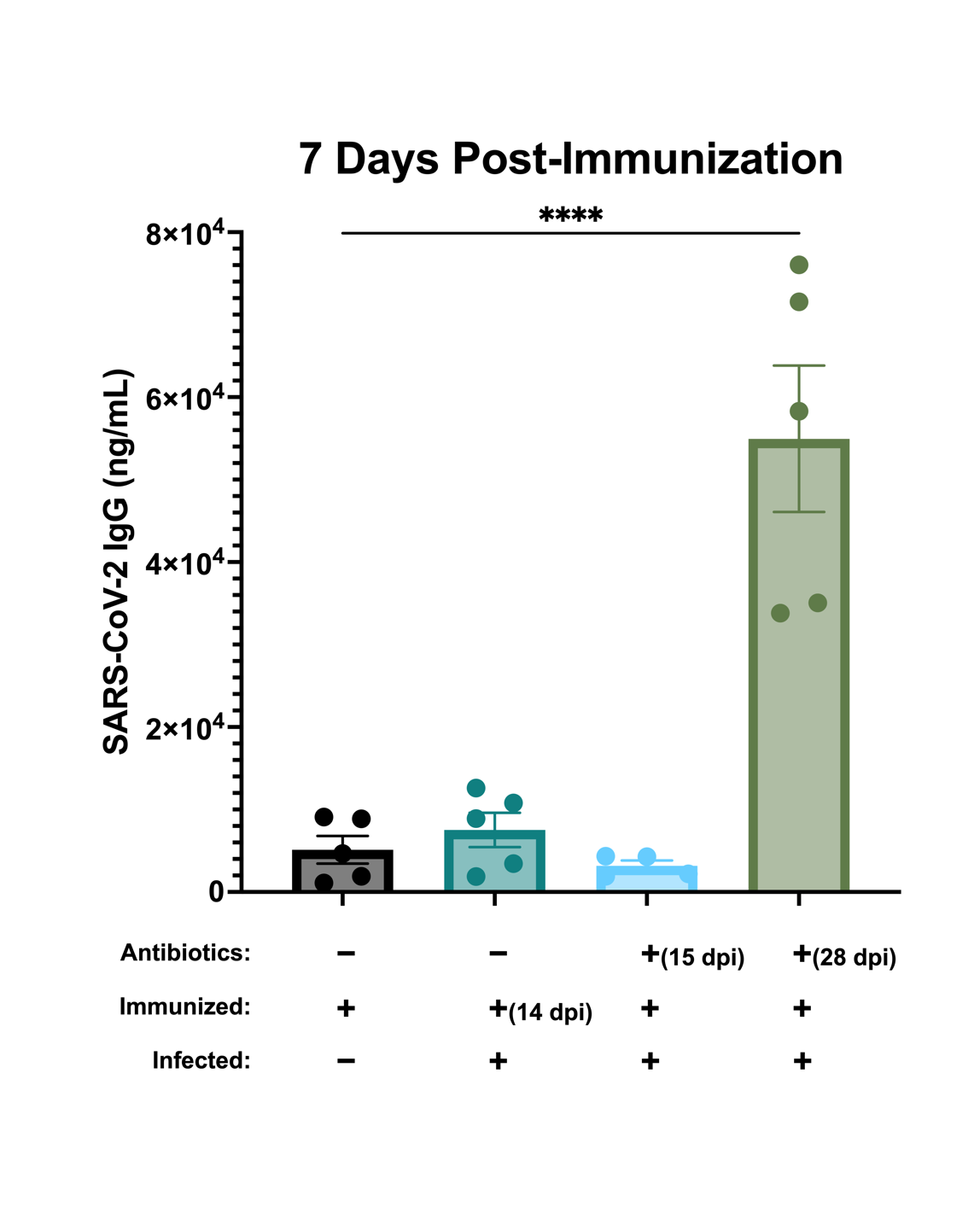


**Figure S2.** IgG response ± SEM to unrelated antigen in uninfected, *B. burgdorferi*-infected, and *B. burgdorferi*-infected antibiotic-treated groups 7 days post-immunization. Post-infection timepoints for immunization and antibiotic treatment are denoted when applicable. Shown are differences between the uninfected and immunized group, *B. burgdorferi*-infected groups that were immunized following antibiotic treatment, and the *B. burgdorferi*-infected but untreated group that was immunized 14 dpi. Significance determined by one-way ANOVA followed by Dunnett’s post-hoc test (****p$\leq$0.0001).


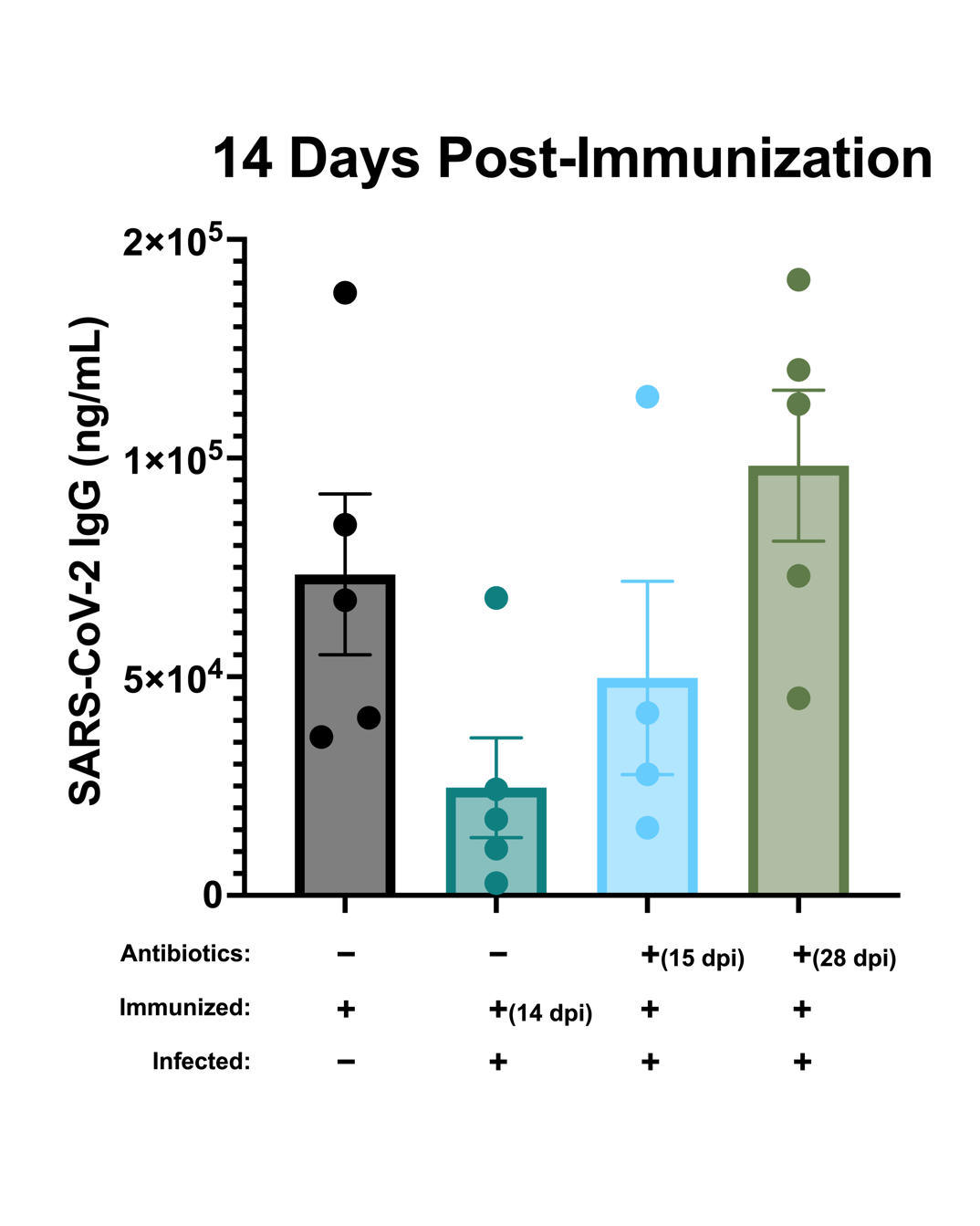


**Figure S3.** IgG response ± SEM to unrelated antigen in uninfected, *B. burgdorferi*-infected, and *B. burgdorferi*-infected antibiotic-treated groups 14 days post-immunization. Post-infection timepoints for immunization and antibiotic treatment are denoted when applicable. Shown are differences between the uninfected and immunized group, *B. burgdorferi*-infected groups that were immunized following antibiotic treatment, and the *B. burgdorferi*-infected but untreated group that was immunized 14 dpi. Significance determined by one-way ANOVA followed by Dunnett’s post-hoc test.


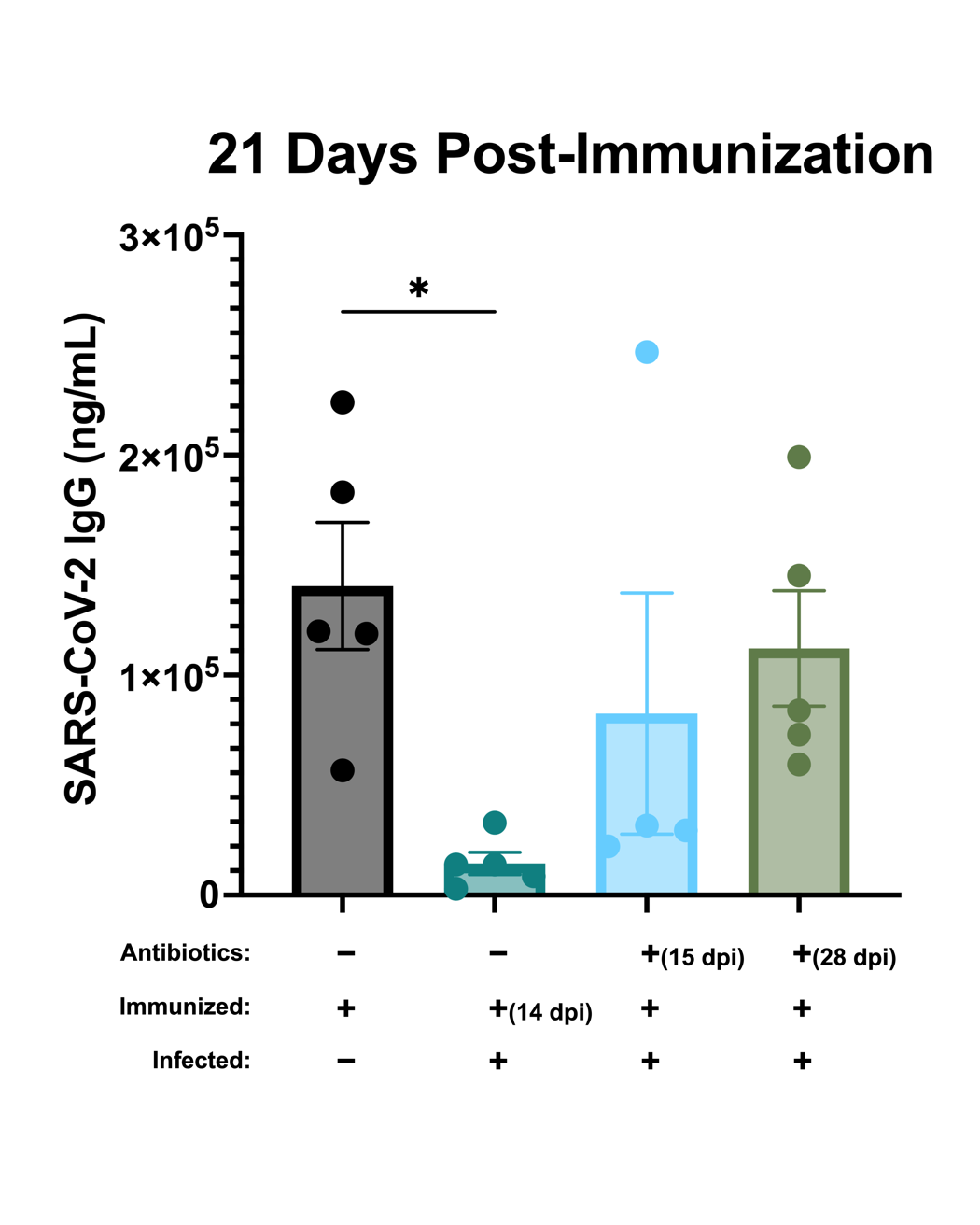


**Figure S4.** IgG response ± SEM to unrelated antigen in uninfected, *B. burgdorferi*-infected, and *B. burgdorferi*-infected antibiotic-treated groups 21 days post-immunization. Post-infection timepoints for immunization and antibiotic treatment are denoted when applicable. Shown are differences between the uninfected and immunized group, *B. burgdorferi*-infected groups that were immunized following antibiotic treatment, and the *B. burgdorferi*-infected but untreated group that was immunized 14 dpi. Significance determined by one-way ANOVA followed by Dunnett’s post-hoc test (*p$\leq$0.05).

# Supplementary Tables

|  |  | Culture | | PCR | |
| --- | --- | --- | --- | --- | --- |
| Group | **Mouse #** | **Bladder** | **Heart** | **Right iLN** | **Left iLN** |
| t0 | 1 | + | - | - | + |
|  | 2 | + | + | NT | NT |
|  | 3 | ND | + | + | - |
|  | 4 | + | ND | NT | NT |
|  | 5 | + | - | + | + |
| t14 | 1 | + | ND | - | - |
|  | 2 | + | + | NT | NT |
|  | 3 | + | ND | - | + |
|  | 4 | + | + | NT | NT |
|  | 5 | ND | + | - | + |
| t28 | 1 | + | + | - | - |
|  | 2 | + | + | NT | NT |
|  | 3 | + | ND | + | + |
|  | 4 | + | + | NT | NT |
|  | 5 | + | + | + | - |
| t42 | 1 | + | + | + | - |
|  | 2 | ND | + | NT | NT |
|  | 3 | + | + | + | + |
|  | 4 | + | + | NT | NT |
|  | 5 | + | + | + | + |
| Uninfected | 1 | - | - | NT | NT |
|  | 2 | - | - | NT | NT |
|  | 3 | - | - | NT | NT |
|  | 4 | - | - | NT | NT |
|  | 5 | - | - | NT | NT |
|  |  |  |  |  |  |
| ND = not determined, culture contaminated | | | | | |
| NT = not tested (used for IHC) | | | | | |

**Table S1.** Shown are culture and PCR results for tissues harvested from the 5 groups of mice used in the study (not antibiotic-treated). All tissue samples were collected 28 days post-immunization with the SARS-CoV-2 spike protein and either cultured in BSK-Y media or used for PCR targeting recA.

|  |  | Pre-treatment | Post-treatment | |  |
| --- | --- | --- | --- | --- | --- |
| Group | **Mouse #** | **Ear Punch Culture** | **Bladder Culture** | **Right Joint Culture** | **Serological** |
| 15d antibiotic | 1 | - | - | - | + |
|  | 2 | + | - | - | + |
|  | 3 | - | - | - | + |
|  | 4 | - | - | - | + |
|  | 5 | + | +(14 dpi) | +(14 dpi) | + |
| 28d antibiotic | 1 | + | - | - | + |
|  | 2 | + | - | - | + |
|  | 3 | + | - | - | + |
|  | 4 | + | - | - | + |
|  | 5 | + | - | - | + |

**Table S2.** Shown are culture and PCR results for tissues harvested from the 2 antibiotic-treated groups of mice used in this study. Ear punches were collected the day before the start of antibiotic treatment to confirm infection. Results from this were variable in the 15d group likely due to different rates of dissemination from the injection site to the ear tissue. Mouse #5 in the 15d antibiotic group was euthanized at 14 dpi (denoted above) to confirm infection via bladder and joint culture and therefore could not used in downstream studies with the SARS-CoV-2 immunization. All tissue samples from the remaining mice were collected 28 days post-immunization with the SARS-CoV-2 spike protein and were cultured in BSK-Y media to confirm clearance of the infection following antibiotic treatment.
